# Supplementary material for: Oxygen Sensing Coordinates Photomorphogenesis to Facilitate Seedling Survival
Source: Curr Biol. 2015 Jun 1;25(11):1483–8. doi: 10.1016/j.cub.2015.03.060 (PMC4454774; doi:10.1016/j.cub.2015.03.060)
Supplement: Document S1. Supplemental Experimental Procedures and Figures S1–S3 [file mmc1.pdf]

Current Biology
Supplemental Information

Oxygen Sensing Coordinates Photomorphogenesis to Facilitate Seedling Survival

Mohamad Abbas, Sophie Berckhan, Daniel Rooney, Daniel J. Gibbs, Jorge Vicente Conde, Cristina Sousa Correia, George W. Bassel, Nora Marin-de la Rosa, José León, David Alabadí, Miguel A. Blázquez, and Michael J. Holdsworth

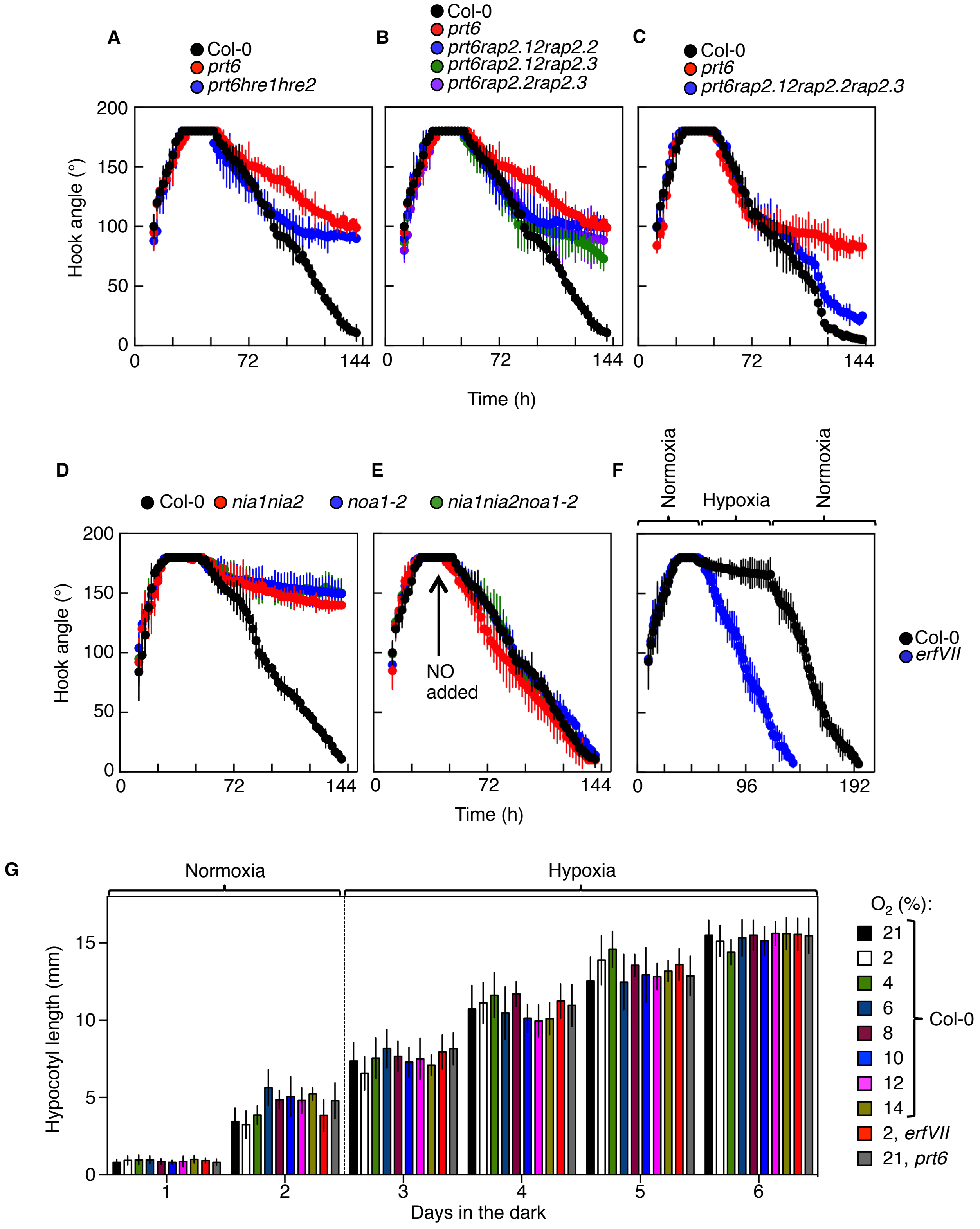
A
Col-0
prt6
prt6hre1hre2
B
Col-0
prt6
prt6rap2.12rap2.2
prt6rap2.12rap2.3
prt6rap2.2rap2.3
C
Col-0
prt6
prt6rap2.12rap2.2rap2.3
Hook angle (°)
200
150
100
50
0
0
72
144
Time (h)
D
E
F
Col-0
nia1nia2
noa1-2
nia1nia2noa1-2
NO added
Normoxia
Hypoxia
Normoxia
Col-0
erfVII
96
192
G
Normoxia
Hypoxia
Hypocotyl length (mm)
15
10
5
0
1
2
3
4
5
6
Days in the dark
O2 (%):
21
2
4
6
8
10
12
14
Col-0
2, erfVII
21, prt6

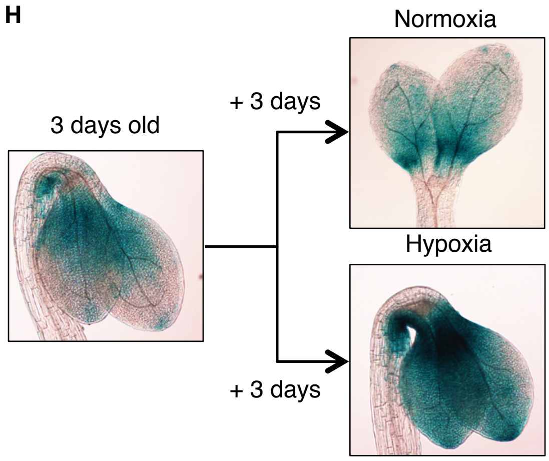

Figure S1, Related to Figure 2. Group VII ERFs function to control apical hook opening.
A,B,C. Comparison of apical hook opening of WT (Col-0), *prt6* and *prt6* in combination with increasing numbers of null mutated *erfVII* genes. The lowest order mutant combination that can open its hook is *prt6rap2.12rap2.2rap2.3*.
D. NO synthesis is required for hook opening. Mutants with reduced NO synthesis, *nia1nia2, noa1-2, nia1nia2noa1-2* all show maintenance of the apical hook in normoxia compared to WT (Col-0).
E. Application of NO gas (70ppm) removes the hook maintenance phenotype of *nia1nia2, noa1-2* and *nia1nia2noa1-2.*
F. WT (Col-0) seedling hooks remain closed during extended hypoxia but open rapidly on transfer to normoxia. *erfVII* hooks open rapidly even in hypoxia.
G. Hypocotyl elongation is not affected by hypoxia in WT and *erfVII*, or normoxia in *prt6*.
H. A gradient of auxin activity is maintained at the apical hook under hypoxia. WT transgenic seedlings expressing the auxin reporter *DR5*::*GUS* were grown for six days in normoxia or transferred after three days to hypoxic conditions. GUS staining was carried out for only four hours because longer staining resulted in very dark blue hooks.

Error bars indicate standard deviation from the mean.

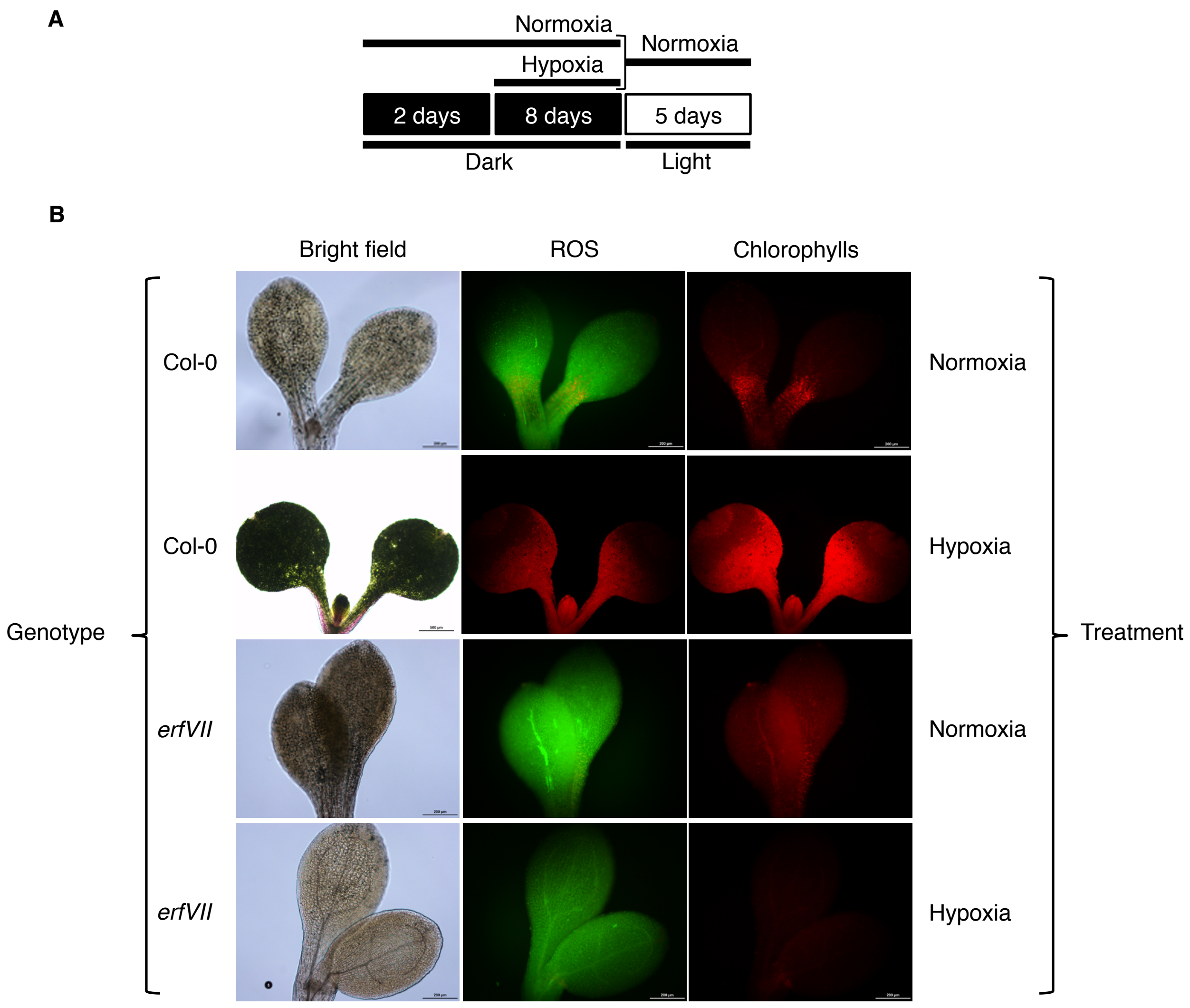

Figure S2, Related to Figure 3. Response of cotyledons to long term growth in the dark.
A. Schematic of experimental design.
B. Analysis of cotyledon phenotypes. Reactive Oxygen Species (ROS) accumulation in cotyledons is shown. Green fluorescence (H_2DCFDA staining) indicates ROS. Chlorophyll auto fluorescence is shown in red, bright field images of seedlings are shown for comparison.

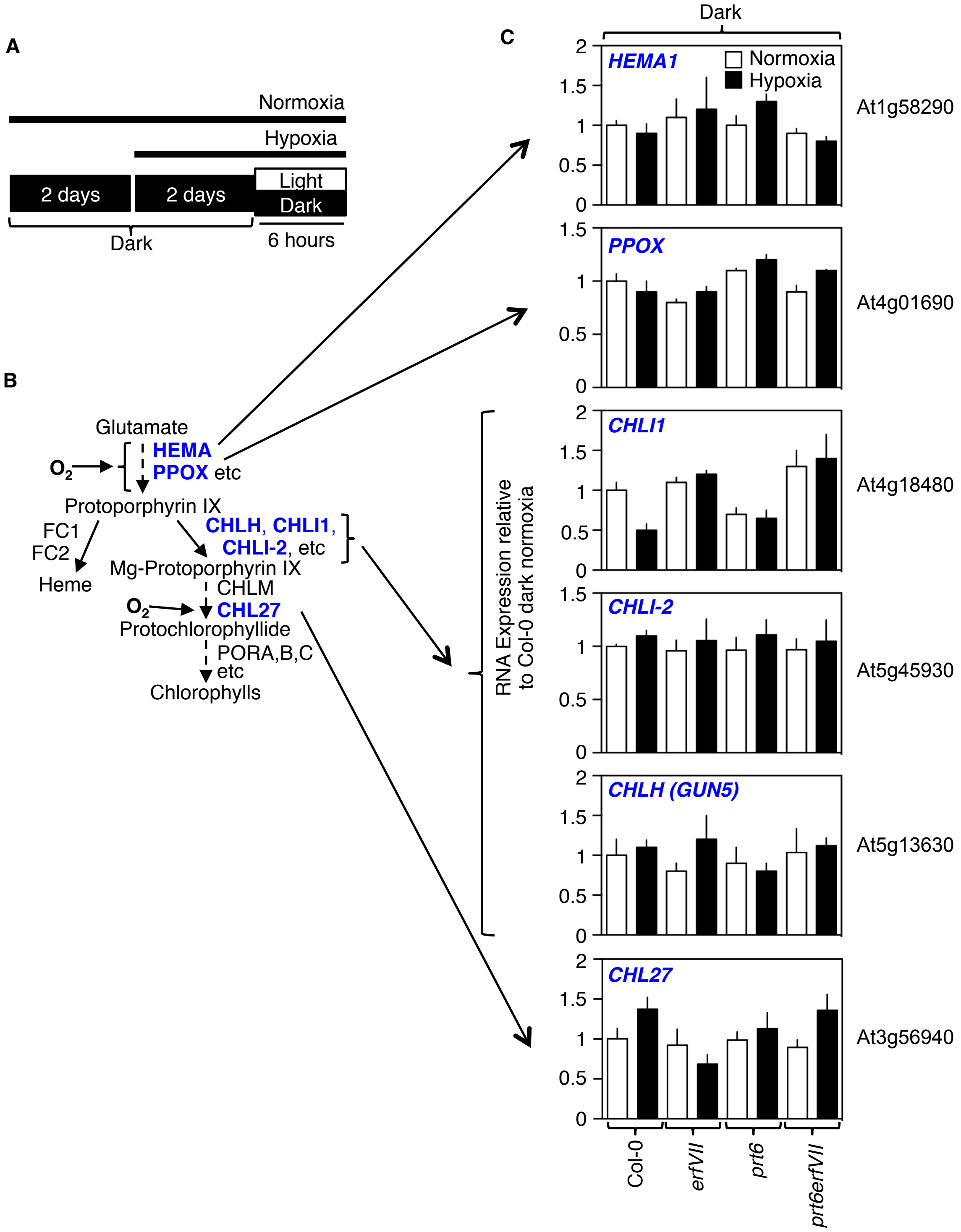

A
Normoxia
Hypoxia
2 days
2 days
Light
Dark
Dark
6 hours
B
Glutamate
O2
HEMA
PPOX etc
Protoporphyrin IX
FC1
FC2
Heme
CHLH, CHLI1, CHLI-2, etc
Mg-Protoporphyrin IX
CHLM
O2
CHL27
Protochlorophyllide
PORA,B,C etc
Chlorophylls
C
Dark
HEMA1
Normoxia
Hypoxia
At1g58290
PPOX
At4g01690
CHLI1
At4g18480
CHLI-2
At5g45930
CHLH (GUN5)
At5g13630
CHL27
At3g56940
RNA Expression relative to Col-0 dark normoxia
Col-0
erfVII
prt6
prt6erfVII

I

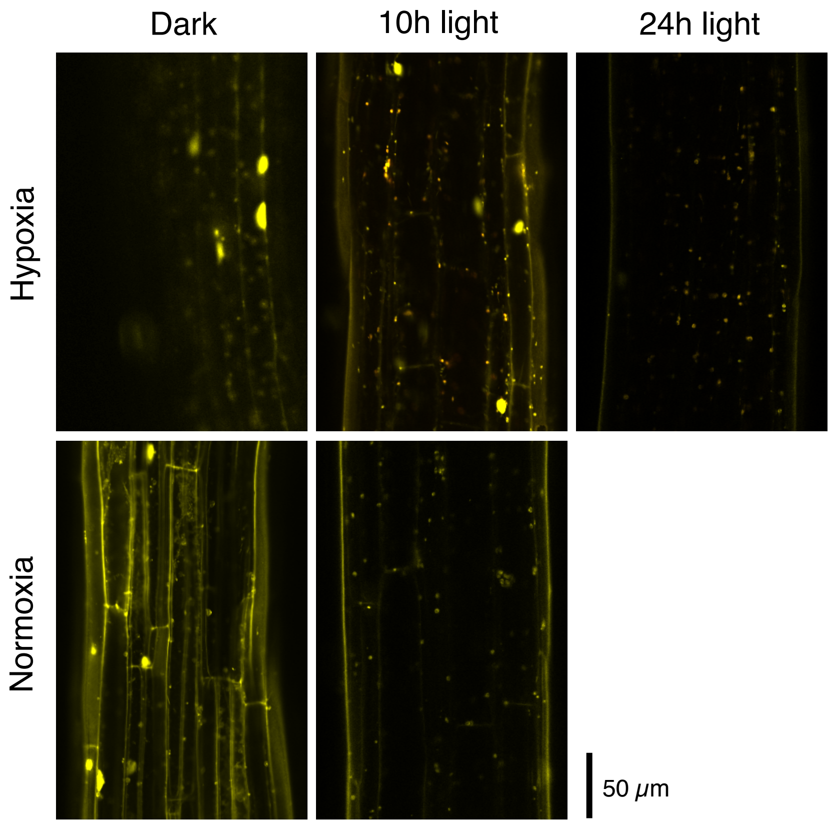

II

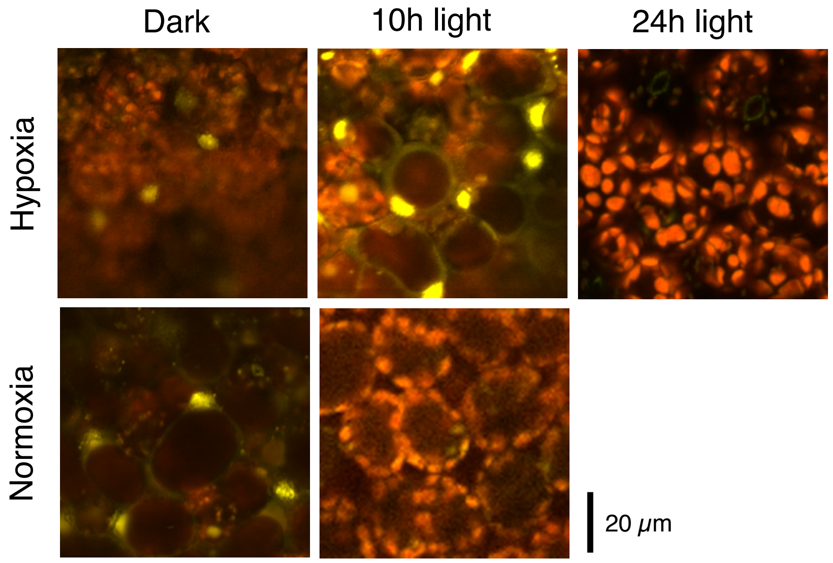

Figure S3, Related to Figure 4. Expression of many tetrapyrrole biosynthesis genes is not affected by hypoxia, and light and hypoxia influence ERFVII subcellular location Quantitative (Q)rtPCR analysis of chlorophyll biosynthesis gene expression in etiolated seedlings of WT (Col-0) and N-end rule pathway mutants grown in normoxia or hypoxia.

A. Experimental setup. Etiolated seedlings were grown for four days in the dark, the final 2 days being in either normoxia or hypoxia, then seedlings were left in the dark (this figure) or exposed to light (Figure 4) for 6 hours.

B. Schematic of the chlorophyll biosynthesis pathway in higher plants. C. QrtPCR of selected genes from 4 day old etiolated seedlings, the final 2 days being in either normoxia or hypoxia.

D. Hypoxia causes delayed in light-regulated removal of nuclear located YFP-RAP2.3 (*35S:YFP-RAP2.3*)
in (I) hypocotyls and (II) cotyledons.

Error bars indicate standard deviation from the mean.

Supplemental Experimental Procedures:

Plant material:

Unless otherwise stated *Arabidopsis thaliana* seeds were obtained from the Nottingham *Arabidopsis* Stock Centre (NASC) and were grown for propagation as previously described [S1]. All mutants are in the Col-0 (Wild Type) accession. Novel reported mutant combinations were identified by PCR (genotyping primers as previously described [S1]). White poppy (*Papaver somniferum* L.) and *Nicotiana benthamiana* Domin. seeds were obtained from Cristina Ferrándiz (IBMCP, Valencia, Spain). A *pENTR* vector containing the *RAP2.3* ORF has been described [S2]. To prepare *35S:YFP-RAP2.3*, the *RAP2.3* entry clone was transferred by a LR reaction to the destination vector *pEarleyGate104* [S3] using the Gateway technology (Invitrogen). Transgenic *Arabidopsis* containing *promERFVII:MA-ERFVII* constructs, using full-length genomic DNA sequence (2 kb upstream of the ATG, finishing at the STOP codon) as PCR template, were produced as previously described for *promRAP2.3:MA-RAP2.3* [S1] using primers described in Table S1, and transformed into *Agrobacterium* (strain GV3101 pMP90) and *Arabidopsis* using standard protocols *[S4]*.

Analysis of seedling growth:

Seeds were plated on half-strength Murashige and Skoog (MS) medium (Duchefa) with 1% sucrose, 1% agar (pH 5.7), chilled for 2 to 4 days at 4°C, and exposed to white fluorescent light (90–100 µmol m^{-2} sec^{-1}) for 8-12 hours at 22°C to initiate germination, after which plates were incubated in darkness, unless indicated in the text. Experiments analyzing the effect of different oxygen levels were conducted as follows; seedlings were placed 48 hours after germination in a transparent

methacrylate container (volume = 22 l) connected to a nitrogen (N_2) cylinder (Linde Industrial Gases, Spain) and an oxygen meter attached to the outlet pipe (KANE250 Compact Flue Gas Analyzer-Kane International Ltd, UK). Nitrogen was flushed into the system until the desired oxygen level was reached, and oxygen levels were monitored at the beginning and end of the experiment. For NO treatments, 70 ppm (final concentration) pure NO (Linde Industrial Gases, Spain) was injected into the container. For analysis of Reactive Oxygen Species (ROS) seedlings were grown in the dark in normoxia or hypoxia and moved into continuous white light (95 µmol m^{-2} s^{-1}) for an additional 4 d. Seedlings were incubated for 30 min at 4°C in 10 µM H_2DCFDA (a ROS-sensitive dye) and then washed with 10 mM MES, 0.1 mM CaCl2, pH 6, for 60 min at 22°C. Dye excitation was at 480 nm; emitted light was detected at 535 to 550 nm with a Nikon Eclipse E600 [S5, 6].

Time-course analysis of apical hook development

Development of seedlings was recorded at 1-hour intervals for 7 days from transfer to dark at 22°C with an infrared light source (850 nm LED) by a spectrum-enhanced camera (Microsoft lifestudio). Angles between the hypocotyl axis and cotyledons were measured with ImageJ (NIH; http://rsb.info.nih.gov/ij) as previously reported [S7]. Fifteen seedlings with synchronized germination were processed. Experiments were repeated at least twice.

Gene expression analysis

RNA extraction, cDNA synthesis, and quantitative RT-PCR (RT-qPCR) have been previously described [S8]. Gene-specific signals were normalized over those of the *EF1a* (AT5G60390). For primers used see table S1. In the case of DR5::GUS, β-

glucuronidase (GUS) staining was performed as described previously [S9]. Seedlings were collected and fixed with 90% cold acetone, washed twice with a solution of 50 mM NaPO4 (pH 7.2), 0.2 % (v/v) Triton X-100 (Sigma), 2 mM Potassium Ferrocyanide and Potassium Ferricyanide (Sigma), then 1 mM X-Gluc (Sigma) was added and seedlings were stained overnight at 37°C then cleared with a series of ethanol washes and with chloral hydrate (Sigma) for 2 days. Images were taken using a Nikon Eclipse E600 scope.

Confocal microscopy

Fresh seedlings were mounted on slides with water. Images were taken using a Zeiss LSM 780 confocal microscope on an Axio Observer (Zeiss, http://www.zeiss.com) with a detection wavelength of 497-532 nm.

Analysis of chlorophyll and protochlorophyllide

Four-day-old dark-grown seedlings were exposed to white light for 6 h of and chlorophyll was extracted in darkness from 100 mg tissue with 1 ml of acetone (80%) at 4°C. The chlorophyll content was calculated using spectrophotometric absorbance (*A*) at wavelengths of 603, 645 and 663 nm and 80% acetone as a control, and shown as milligram of chlorophyll per gram of fresh tissue as follows: Chlorophyll a (mg g^{-1}) = $12.7 \times A_{663} - 2.69 \times A_{645}$; chlorophyll b (mg g^{-1}) = $22.9 \times A_{645} - 4.86 \times A_{663}$: and chlorophyll a+b (mg g^{-1}) = $8.02 \times A_{663} + 20.20 \times A_{645}$. Protochlorophyllide levels were measured as previously described [S10, 11]. Twenty dark-grown seedlings were homogenized in 0.8 ml of ice-cold 90% acetone and 0.1% ammonia, left on ice in darkness for 6 hours, centrifuged for 20 minutes at 4°C, and then relative fluorescence

(excitation at 440 nm; emission between 600-700 nm) was measured at room temperature.

Supplemental References:

S1. Gibbs, D.J., Isa, N.M., Movahedi, M., Lozano-Juste, J., Mendiondo, G.M., Berckhan, S., Marin-de la Rosa, N., Conde, J.V., Correia, C.S., Pearce, S.P., et al. (2014). Nitric Oxide Sensing in Plants Is Mediated by Proteolytic Control of Group VII ERF Transcription Factors. Molecular Cell *53*, 369-379.

S2. Marin-de la Rosa, N., Sotillo, B., Miskolczi, P., Gibbs, D.J., Vicente, J., Carbonero, P., Onate-Sanchez, L., Holdsworth, M.J., Bhalerao, R., Alabadi, D., et al. (2014). Large-Scale Identification of Gibberellin-Related Transcription Factors Defines Group VII ETHYLENE RESPONSE FACTORS as Functional DELLA Partners. Plant Physiol. *166*, 1022-1032.

S3. Earley, K.W., Haag, J.R., Pontes, O., Opper, K., Juehne, T., Song, K.M., and Pikaard, C.S. (2006). Gateway-compatible vectors for plant functional genomics and proteomics. Plant J. *45*, 616-629.

S4. Clough, S.J., and Bent, A.F. (1998). Floral dip: a simplified method for *Agrobacterium*-mediated transformation of *Arabidopsis* thaliana. Plant J. *16*, 735-743.

S5. Achard, P., Renou, J.-P., Berthome, R., Harberd, N.P., and Genschik, P. (2008). Plant DELLAs restrain growth and promote survival of adversity by reducing the levels of reactive oxygen species. Current Biology *18*, 656-660.

S6. Cheminant, S., Wild, M., Bouvier, F., Pelletier, S., Renou, J.-P., Erhardt, M., Hayes, S., Terry, M.J., Genschik, P., and Achard, P. (2011). DELLAs Regulate Chlorophyll and Carotenoid Biosynthesis to Prevent Photooxidative Damage during Seedling Deetiolation in Arabidopsis. Plant Cell *23*, 1849-1860.

S7. Gallego-Bartolome, J., Arana, M.V., Vandenbussche, F., Zadnikova, P., Minguet, E.G., Guardiola, V., Van der Straeten, D., Benkova, E., Alabadi, D., and Blazquez, M.A. (2011). Hierarchy of hormone action controlling apical hook development in Arabidopsis. Plant J. *67*, 622-634.

S8. Frigerio, M., Alabadi, D., Perez-Gomez, J., Garcia-Carcel, L., Phillips, A.L., Hedden, P., and Blazquez, M.A. (2006). Transcriptional regulation of gibberellin metabolism genes by auxin signaling in arabidopsis. Plant Physiol. *142*, 553-563.

S9. Zadnikova, P., Petrasek, J., Marhavy, P., Raz, V., Vandenbussche, F., Ding, Z., Schwarzerova, K., Morita, M.T., Tasaka, M., Hejatko, J., et al. (2010). Role of PIN-mediated auxin efflux in apical hook development of Arabidopsis thaliana. Development *137*, 607-617.

S10. Goslings, D., Meskauskiene, R., Kim, C.H., Lee, K.P., Nater, M., and Apel, K. (2004). Concurrent interactions of heme and FLU with Glu tRNA reductase (HEMA1), the target of metabolic feedback inhibition of tetrapyrrole biosynthesis, in dark- and light-grown Arabidopsis plants. Plant J. *40*, 957-967.

S11. Zhong, S.W., Shi, H., Xue, C., Wei, N., Guo, H.W., and Deng, X.W. (2014). Ethylene-orchestrated circuitry coordinates a seedling's response to soil cover and etiolated growth. PNAS (USA) *111*, 3913-3920.
