# Supplementary material for: Oxygen Sensing Coordinates Photomorphogenesis to Facilitate Seedling Survival
Source: Curr Biol. 2015 Jun 1;25(11):1483–8. doi: 10.1016/j.cub.2015.03.060 (PMC4454774; doi:10.1016/j.cub.2015.03.060)
Supplement: Document S2. Article plus Supplemental Information [file mmc3.pdf]

Report

Current Biology

Oxygen Sensing Coordinates Photomorphogenesis to Facilitate Seedling Survival

Highlights

- Low oxygen (hypoxia) enhances seedling survival in the dark
- Hypoxia is sensed by oxygen-mediated degradation of ERFVII transcription factors
- Stable ERFVIIs enhance dark-activated development and repress light-activated development
- Plants monitor the gaseous environment to coordinate photomorphogenesis

Authors

Mohamad Abbas, Sophie Berckhan, ..., Miguel A. Blázquez, Michael J. Holdsworth

Correspondence

mblazquez@ibmcp.upv.es (M.A.B.), michael.holdsworth@nottingham.ac.uk (M.J.H.)

In Brief

Abbas et al. demonstrate that hypoxia sensing is an important component of photomorphogenesis. Hypoxia acts as a positive external positional cue controlling morphogenetic and biochemical adaptations to protect the stem cell niche. Sensing hypoxia in the dark coordinates responses that maximize survival and establishment in the light.

Abbas et al., 2015, Current Biology *25*, 1483–1488
June 1, 2015 ©2015 The Authors
http://dx.doi.org/10.1016/j.cub.2015.03.060

CrossMark

CellPress

Current Biology
Report

Oxygen Sensing Coordinates Photomorphogenesis to Facilitate Seedling Survival

Mohamad Abbas,[1] Sophie Berckhan,[2] Daniel J. Rooney,[2] Daniel J. Gibbs,[2,3] Jorge Vicente Conde,[2] Cristina Sousa Correia,[2] George W. Bassel,[2,3] Nora Marín-de la Rosa,[1] José León,[1] David Alabadí,[1] Miguel A. Blázquez,[1,*] and Michael J. Holdsworth[2,*]
[1]Instituto de Biología Molecular y Celular de Plantas, Consejo Superior de Investigaciones Científicas, Universidad Politécnica de Valencia, Ciudad Politécnica de la Innovación, 46022 Valencia, Spain
[2]Division of Plant and Crop Sciences, School of Biosciences, University of Nottingham, Loughborough LE12 5RD, UK
[3]School of Biosciences, University of Birmingham, Edgbaston B15 2TT, UK
*Correspondence: mblazquez@ibmcp.upv.es (M.A.B.), michael.holdsworth@nottingham.ac.uk (M.J.H.)
http://dx.doi.org/10.1016/j.cub.2015.03.060
This is an open access article under the CC BY license (http://creativecommons.org/licenses/by/4.0/).

SUMMARY

Successful emergence from the soil is essential for plant establishment in natural and farmed systems. It has been assumed that the absence of light in the soil is the preeminent signal perceived during early seedling development, leading to a distinct morphogenic plan (skotomorphogenesis) [1], characterized by traits providing an adaptive advantage until emergence and photomorphogenesis. These traits include suppressed chlorophyll synthesis, promotion of hypocotyl elongation, and formation of a closed apical hook that protects the stem cell niche from damage [2, 3]. However, absence of light by itself is not a sufficient environmental signal for early seedling development [4, 5]. Reduced oxygen levels (hypoxia) can occur in water-logged soils [6–8]. We therefore hypothesized that below-ground hypoxia may be an important, but thus far undiscovered, ecological component regulating seedling development. Here, we show that survival and establishment of seedlings following darkness depend on their ability to sense hypoxia, through enhanced stability of group VII Ethylene Response Factor (ERFVII) transcription factors. Hypoxia is perceived as a positive environmental component in diverse taxa of flowering plants, promoting maintenance of skotomorphogenic traits. Hypoxia greatly enhances survival once light is perceived, while oxygen is necessary for the subsequent effective completion of photomorphogenesis. Together with light perception, oxygen sensing therefore allows an integrated response to the complex and changing physical microenvironment encountered during early seedling growth. We propose that plants monitor the soil's gaseous environment after germination, using hypoxia as a key external cue to protect the stem cell niche, thus ensuring successful rapid establishment upon emergence above ground.

RESULTS AND DISCUSSION

We analyzed the effect of oxygen availability and light on seedling growth following germination in species representing distinct branches of eudicot phylogeny: *Papaver somniferum*, *Nicotiana benthamiana*, and *Arabidopsis thaliana* (*Arabidopsis*). Apical hook development is characterized by three phases: formation, maintenance, and opening [5, 9]. Contrary to the observation that seedlings kept in darkness under normal oxygen levels (normoxia) eventually open the hook (Figure 1A), we found that hypoxic conditions (2% oxygen imposed following the hook maintenance phase) strongly inhibited opening in all species, which was reverted following transfer back to normoxia (Figures 1A and 1B). The response of final hook angle to increasing oxygen tensions in *Arabidopsis* is linear, suggesting stochastic cumulative sensing and response to oxygen (Figure 1C) [10]. Etiolated seedlings maintained under hypoxia were able to complete hook opening when exposed to light but had defective unfolding and greening of cotyledons, indicating that oxygen is required for the complete response to light (Figure 1D). Oxygen sensing in flowering plants is therefore a major component of skotomorphogenic development and the transition to photomorphogenesis.

Oxygen is sensed in plants by the Cysteine (Cys) branch of the Arginine (Arg)/N-end rule pathway of targeted proteolysis, using group VII Ethylene Response Factors (ERFVIIs) as substrates [11, 12] (Figure 2A). The N-end rule pathway relates the in vivo stability of a protein to the nature of its N terminus, which may be stabilizing or destabilizing (the N-degron) [14]. In normoxia, ERFVIIs are destabilized through oxidation of N-terminal (Nt)-Cys, which targets the proteins for degradation via the N-end rule pathway. Under hypoxia, Nt-Cys is not oxidized and substrates are stable, enhancing growth and development [11–13]. This mechanism is also used to sense nitric oxide (NO), an essential component of Nt-Cys oxidation [15, 16]. There are five ERFVIIs in *Arabidopsis*: RELATED TO AP (RAP)2.12, RAP2.2, RAP2.3, HYPOXIA RESPONSIVE (HRE)1, and HRE2 [17].

We investigated whether early seedling growth in *Arabidopsis* is controlled by oxygen sensing through the N-end rule pathway. We analyzed apical hook development in mutant seedlings lacking either E3 ligase (PRT6) or Arginyl-transferase (ATE) functions

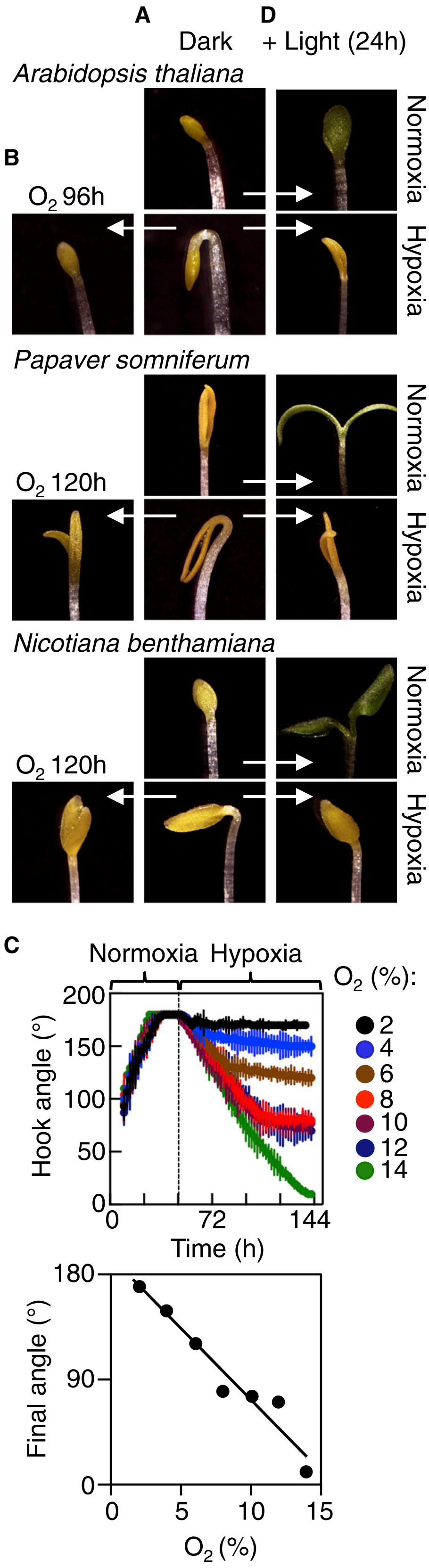

Figure 1. Skotomorphogenic Traits Are Regulated by Oxygen Sensing in Flowering Plants

(A) Images of final apical hook angle of etiolated seedlings of *Arabidopsis thaliana*, *Papaver somniferum*, and *Nicotiana benthamiana* in normoxia or hypoxia (168 hr, 168 hr, and 144 hr in the dark, respectively). Hypoxia treatment was continuously applied following initiation of the hook maintenance phase.

(B) Response of the apical hook after transfer from hypoxia (A) to normoxia for the indicated times.

(C) Response of the apical hook angle of *Arabidopsis* to increasing oxygen levels.

(D) Images of cotyledon greening of seedlings following transfer to light under normoxia or hypoxia (24 hr).

Error bars indicate SD from the mean.

(Figure 2A). In these mutants, substrates of the Cys-Arg/N-end rule pathway are constitutively stable, but with different N termini. In contrast to wild-type (WT; accession Col-0) etiolated seedlings, apical hooks of *prt6* and *ate1 ate2* did not fully open in the dark under normoxia (Figure 2B). Remarkably, the *prt6 rap2.12 rap2.2 rap2.3 hre1 hre2* sextuple mutant (*prt6 erfVII*), which lacks the function of all five ERFVIIs, reverted the *prt6* phenotype (Figures 2B and S1), indicating that ERFVIIs act redundantly to repress hook opening. Opening of apical hooks was also inhibited in NO-deficient mutants, a phenotype reverted in the *nia1 nia2 erfVII* mutant or by treatment with NO (Figures 2C and S1). To confirm that ERFVIIs are an integral component of oxygen sensing controlling early seedling development, we tested the ability of individual ERFVIIs to repress hook opening by expressing mutant stable versions (in which Cys-2 was changed to Alanine, a stabilizing residue; Figure 2A) driven by their endogenous promoters (*promERFVII:MA-ERFVII*) providing MA-ERFVII protein. All five MA-ERFVIIs were able to inhibit hook opening compared to WT, indicating that Cys-Arg-N-end rule-mediated degradation of all ERFVIIs contributes to hook opening (Figure 2D). In order to provide unequivocal evidence that oxygen sensing by ERFVIIs controls hook opening, we analyzed hook development in WT and *rap2.12 rap2.2 rap2.3 hre1 hre2* (*erfVII*) pentuple mutant (which lacks all ERFVII activity) seedlings under conditions where hypoxia was imposed at the end of the hook maintenance phase (Figure 2E). Under hypoxia, WT etiolated seedlings were completely unable to open their hooks. This, however, was not the result of a loss of respiratory energy but a specific consequence of oxygen sensing, as *erfVII* mutant seedlings completed hook opening under hypoxia with similar kinetics to WT seedlings under normoxia. In addition, return to normoxia following a prolonged hypoxic treatment allowed hooks of WT seedlings to open (Figure S1), reaffirming a role for a fast-responding oxygen-sensing mechanism. We observed no effect of hypoxia on hypocotyl elongation, demonstrating that the observed phenotypes are not a consequence of hypoxia-induced quiescence (Figure S1). Apical hook development has been intimately linked to the dynamics of auxin levels [2]. Accordingly, we observed a strong correlation between the presence of a gradient of auxin activity across the apical hook and the maintenance of the hook under hypoxia (Figure S1), suggesting oxygen-mediated regulation of localized auxin responses.

Given that oxygen is required for complete response to light (Figure 1D), we investigated whether greening of cotyledons,

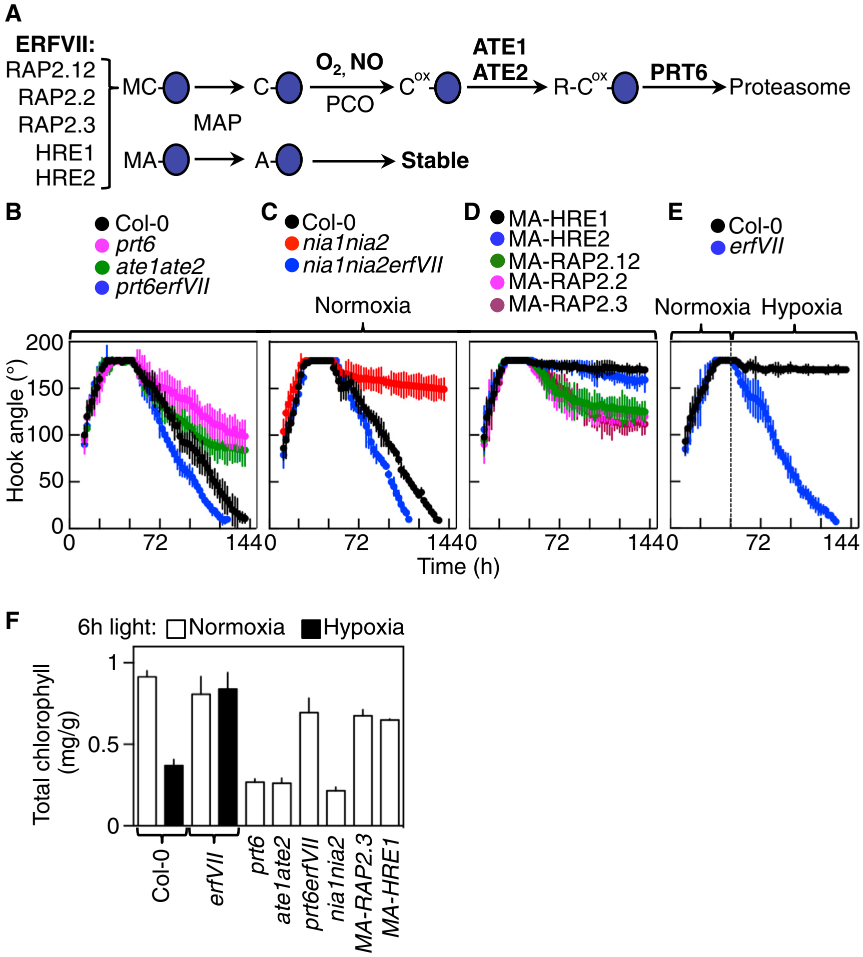

Figure 2. Oxygen Sensing during Skotomorphogenesis and Photomorphogenesis Is Controlled by ERFVII Transcription Factors and the N-End Rule Pathway

(A) Schematic of the Cys-Arg/N-end rule pathway. Single amino acid abbreviations are used. ox, oxidized; NO, nitric oxide; PCO, plant-specific Plant Cysteine Oxidase [13]; MAP, Methionine Amino-Peptidase; ATE Arginyl Transferase; PRT6, PROTEOLYSIS E3 ligase. Protein substrates are indicated as blue ovals.

(B–E) Dynamics of apical hook angle for etiolated seedlings of WT (Col-0), N-end rule pathway and NO synthesis mutants, and transgenics, containing stabilized ERFVIIs expressed under endogenous promoters (*promERFVII:MA-ERFVII*).

(F) Chlorophyll content of 4-day-old seedlings transferred to the light for 6 hr.

Error bars indicate SD from the mean.

another key component of photomorphogenesis, is also mediated by oxygen-dependent ERFVII degradation. Etiolated seedlings of WT and N-end rule mutants were analyzed following transfer to light under hypoxic or normoxic conditions. After transfer to light under normoxia, total chlorophyll levels were much lower in *prt6*, *ate1 ate2*, and *nia1 nia2* mutants compared to WT, but similar in *prt6 erfVII* (Figure 2F). Chlorophyll levels were also reduced in WT seedlings under hypoxia following transfer to light, but not in *erfVII*. Individual stable ERVIIs MA-HRE1 and MA-RAP2.3 driven by their endogenous promoters were not able to repress chlorophyll accumulation, indicating that they act in combination, perhaps as part of heteromeric complexes, with other ERFVIIs, unlike their roles in apical hook maintenance.

Our results indicate that oxygen availability is sensed by ERFVIIs during early seedling development and prompt the hypothesis that low oxygen levels would also enhance seedling survival during exposure to prolonged darkness. As previously reported [4], we found that after extended exposure to dark under normoxia, WT seedlings died, whereas here we show that hypoxia allowed complete recovery of cotyledon greening and even the capacity for primary leaf growth (Figures 3A, 3B, and 3C). Hypoxia-mediated survival was completely dependent on ERFVIIs (Figures 3B and 3C), which restricted the accumulation of reactive oxygen species (ROS) (Figure S2). As a confirmation for the involvement of ERFVIIs in survival after long exposure to darkness, only *prt6*, but not *prt6 erfVII*, seedlings were able to survive long-term exposure to dark normoxia (Figure 3D). Photo-oxidative damage caused by high ROS levels has been linked to excessive accumulation of protochlorophyllide (PC) in darkness [4]; therefore, observed reduced levels of PC in *prt6* mutants compared with WT, *erfVII* and *prt6 erfVII* (Figure 3E), are the likely explanation for the enhanced survival. These results demonstrate that stabilized ERFVIIs protect seedlings from prolonged exposure to dark conditions and permit subsequent growth of the apical meristem following transfer to light.

In darkness, chlorophyll biosynthesis is known to be repressed by PIF transcription factors [18]. However, our analysis of PC accumulation suggested that ERFVIIs repress expression of chlorophyll biosynthesis genes in low-oxygen environments. We therefore analyzed the influence of light and hypoxia on mRNA transcript accumulation for enzymes of tetrapyrrole synthesis (Figures 4A–4D). For several genes, expression in etiolated seedlings was not influenced by hypoxia (Figure S3). However, expression of the chloroplastic form of heme synthase, *FC2*, and *CHLM*, *PORA*, *PORB*, *PORC GUN1*, and *GUN4* was greatly repressed in WT by hypoxia both in the dark and, following transfer, in the light, and was also constitutively repressed in *prt6* independent of oxygen availability (Figures 4C and 4D). Furthermore, this repression was not observed in *erfVII* or *prt6 erfVII* mutants, indicating that downregulation is achieved by stabilized ERFVIIs. In contrast, *FC1* (encoding chloroplastic and mitochondrial heme synthase [19]) expression was enhanced under hypoxia, in an ERFVII-dependent manner (Figure 4D). Our results suggest a homeostatic mechanism whereby hypoxia-stabilized ERFVIIs repress several steps of the oxygen-requiring tetrapyrrole pathway. Together, these

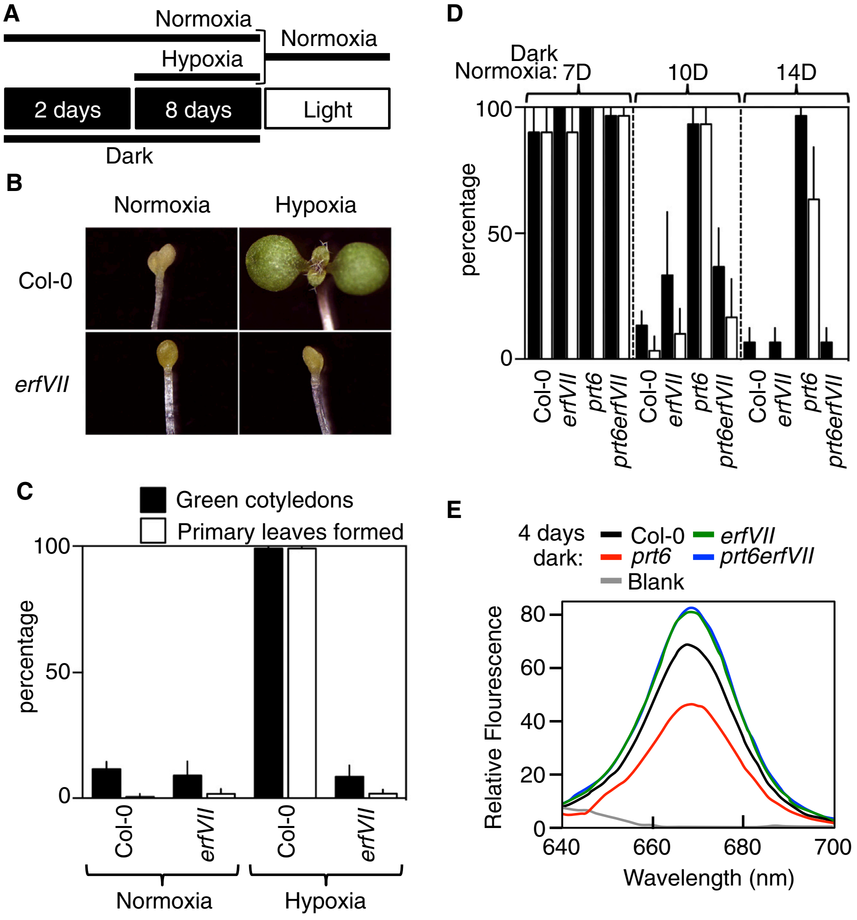

Figure 3. Stabilized ERFVIIs Enhance Long-Term Survival in the Dark

(A) Schematic of experimental design to analyze the effect of long-term development in the dark followed by transfer to the light.

(B and C) Quantification and images of cotyledon greening and primary leaf expansion in WT and *erfVII* seedlings in response to light following exposure to dark under hypoxic or normoxic conditions.

(D) Quantification of cotyledon greening and primary leaf expansion in WT and mutant seedlings in response to light following exposure to increased periods of dark under normoxic conditions.

(E) Relative fluorescence of protochlorophyllide in 4-day etiolated seedlings.

Error bars indicate SD from the mean.

results demonstrate an important role for oxygen sensing in chlorophyll biosynthesis by N-end rule control of ERFVII stability.

The observation that in the light chlorophyll biosynthesis is not completely impaired in hypoxia suggests light either compensates for repression of chlorophyll biosynthesis by ERFVIIs or promotes inactivation of ERFVIIs. It has been shown that cytosolic RAP2.12 moves to the nucleus in response to the hypoxic signal [12]. In agreement with this result, we found that in etiolated seedlings under hypoxia, the subcellular location of constitutively expressed stabilized RAP2.3 (*35S:YFP-RAP2.3*) changed from cytoplasm and nucleus to exclusively nucleus (Figures 4E and S3). However, under normoxia and following transfer to light, this stabilized ERFVII is removed from the nucleus, through a mechanism unrelated to the ERFVII N-degron, as Nt-Cys is not present in the analyzed protein due to the Nt-YFP fusion. However, it is interesting to note that this degradation occurs much more slowly in hypoxia. These results show that light can override ERFVII function even under hypoxia, eventually becoming the dominant environmental signal.

In addition to its metabolic requirement for aerobic respiration, higher eukaryotes use specific sensing of molecular oxygen as a mechanism to control physiology and development [20, 21]. Animals use a different Hypoxia-Inducible Factor (HIF) system to sense oxygen [20]. It has been shown that embryos of *Caenorhabditis elegans* exhibit diapause (arrested development) in response to hypoxia, controlled by maternal nonautonomous expression of neural HIF-1 activity [22]. The rice *Submergence1A* (*Sub1A*) locus (encoding an ERFVII) provides tolerance to submergence-induced hypoxia through a quiescence strategy [21], and work reported here demonstrates that etiolated seedlings of higher plants restrict photomorphogenic development under low-oxygen stress and survive extended low-oxygen conditions through stabilization of ERFVIIs. Thus, morphogenetic and biochemical adaptations to survive hypoxic environments in animals and plants may share common features.

Our data demonstrate that oxygen sensing, in addition to light perception, is a key component of early seedling development, functioning to protect the apical meristem stem cell niche and prevent photo-oxidative damage. We show that long-term survival of seedlings in the dark depends on their ability to sense oxygen and that counter-intuitively hypoxic conditions are an important environmental component enhancing long-term seedling survival. In addition to previous work demonstrating a role for ERFVIIs in plant responses to waterlogging and hypoxia, our current work highlights how sensing of the gaseous environment may play a more general role in plant growth and development. Low light and high ethylene production are similarities between the phenomenologies of flooding and skotomorphogenesis [23, 24]. However, the response of etiolated seedlings described here is not related directly to escape or quiescence strategies associated with long-term submergence [21] but to rapid and non-permanent hypoxic conditions that could occur, for example, after heavy rain in soil with good draining capacity. In agreement with this hypothesis, oxygen sensing does not appear to influence one skotomorphogenic trait, hypocotyl elongation, as etiolated *prt6* seedlings, and WT and *erfVII* seedlings under hypoxia, elongate hypocotyls similarly to WT under normoxia [15] (Figure S1), suggesting that oxygen sensing is specifically related to protection of the apical meristem stem cell niche.

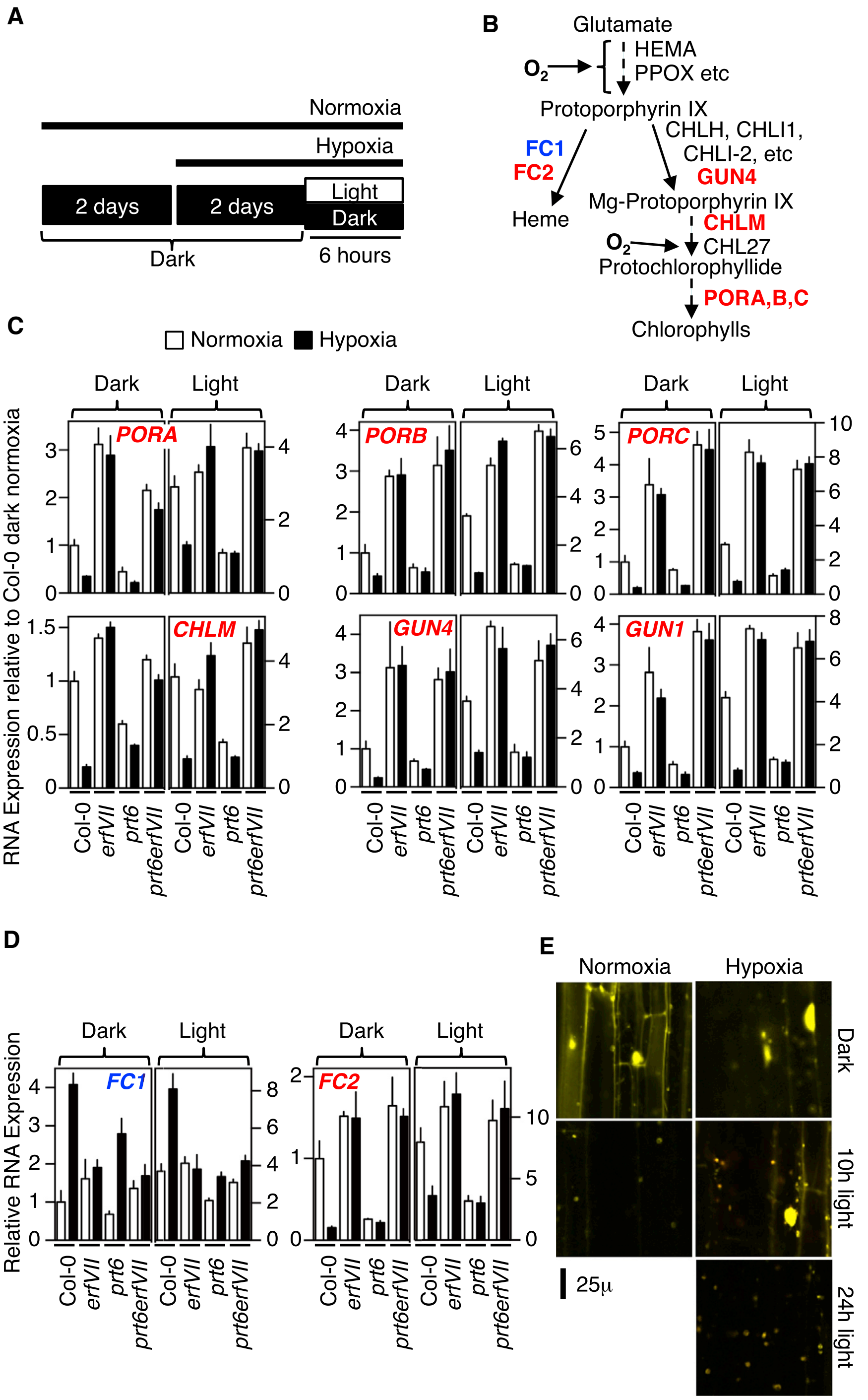

Figure 4. Control of Tetrapyrrole Synthesis Gene Expression by Oxygen and ERFVIIs

(A) Schematic of experimental design. Etiolated seedlings were grown for 4 days in the dark, the final 2 days being in either normoxia or hypoxia. Then, seedlings were left in the dark or exposed to light for 6 hr.

(B) Diagram of chlorophyll biosynthesis pathway. Enzymes are shown next to intermediate compounds of the pathway. Oxygen (O_2)-dependent sections of the pathway are indicated.

(C and D) Expression of tetrapyrrole and heme synthesis genes in WT and mutant lines in response to hypoxia and light.

(E) Changes in subcellular location and abundance of constitutively expressed YFP-RAP2.3 protein in response to oxygen and light.

Error bars indicate SD from the mean.

Recently it was shown that oxygen acts as an internal developmental positional cue in plants [25]; in this work, we have shown that it also acts as an environmental positional cue. The underground environment combines both oxygen and light gradients. We suggest that whereas PIF function integrates responses to light in early seedling growth [26, 27], ERFVII function integrates responses to the gaseous environment, and the extent of overlap between the two pathways remains to be determined. Oxygen sensing may therefore provide an adaptive advantage to seedlings growing through the soil, allowing changes in the gaseous atmosphere to be sensed prior to the irreversible transition to photomorphogenic growth. Ultimately, removal of ERFVII-repressive function is assured when seedlings reach the soil surface through oxygen and NO-mediated destruction. Together with light perception, this allows an integrated response to the complex and changing physical microenvironment encountered by the growing etiolated seedling as it struggles through the soil to reach the surface.

SUPPLEMENTAL INFORMATION

Supplemental Information includes Supplemental Experimental Procedures, three figures, and one table and can be found with this article online at http://dx.doi.org/10.1016/j.cub.2015.03.060.

AUTHOR CONTRIBUTIONS

M.J.H., M.A.B., D.A., J.L., M.A., G.W.B., and S.B. conceived and designed experiments. M.A., S.B., J.V.C., D.J.G., D.R., C.S.C., G.W.B., N.M-d.l.R., J.L., D.A., and M.J.H. performed the experiments. M.J.H., M.A.B., D.A., J.L., and M.A. analyzed the data. M.J.H and M.A.B wrote the manuscript.

ACKNOWLEDGMENTS

M.J.H., D.J.G., J.V.C., and C.S.C. were supported by BBSRC grants BB/G010595/1 and BB/K000144/1 (including financial support from SABMiller). G.W.B. was supported by a Marie Curie International Incoming Fellowship. S.B. was supported by a University of Nottingham PhD fellowship. D.R. was supported by a BBSRC DTP PhD fellowship. J.L. and M.A.B., D.A., and M.A. were supported, respectively, through grants from MICINN (Spain) BIO2011-27526 and BIO2010-15071. We thank Christian Fankhauser and François Parcy for comments on the manuscript.

Received: February 3, 2015
Revised: March 11, 2015
Accepted: March 31, 2015
Published: May 14, 2015

REFERENCES

1. Wu, S.H. (2014). Gene expression regulation in photomorphogenesis from the perspective of the central dogma. Annu. Rev. Plant Biol. *65*, 311–333.
2. Abbas, M., Alabadí, D., and Blázquez, M.A. (2013). Differential growth at the apical hook: all roads lead to auxin. Front. Plant Sci. *4*, 441.
3. Mazzella, M.A., Casal, J.J., Muschietti, J.P., and Fox, A.R. (2014). Hormonal networks involved in apical hook development in darkness and their response to light. Front. Plant Sci. *5*, 52.
4. Sperling, U., van Cleve, B., Frick, G., Apel, K., and Armstrong, G.A. (1997). Overexpression of light-dependent PORA or PORB in plants depleted of endogenous POR by far-red light enhances seedling survival in white light and protects against photooxidative damage. Plant J. *12*, 649–658.
5. Raz, V., and Ecker, J.R. (1999). Regulation of differential growth in the apical hook of Arabidopsis. Development *126*, 3661–3668.
6. Crawford, R.M.M. (1992). Oxygen availability as an ecological limit to plant distribution. In Advances in Ecological Research, *Volume 23*, M. Begon, and A.H. Fitter, eds. (Academic Press), pp. 93–185.
7. Liptzin, D., Silver, W.L., and Detto, M. (2011). Temporal dynamics in soil oxygen and greenhouse gases in two humid tropical forests. Ecosystems *14*, 171–182.
8. Silver, W.L., Lugo, A.E., and Keller, M. (1999). Soil oxygen availability and biogeochemistry along rainfall and topographic gradients in upland wet tropical forest soils. Biogeochemistry *44*, 301–328.
9. Gallego-Bartolomé, J., Arana, M.V., Vandenbussche, F., Zádníková, P., Minguet, E.G., Guardiola, V., Van Der Straeten, D., Benkova, E., Alabadí, D., and Blázquez, M.A. (2011). Hierarchy of hormone action controlling apical hook development in Arabidopsis. Plant J. *67*, 622–634.
10. Kosmacz, M., Parlanti, S., Schwarzländer, M., Kragler, F., Licausi, F., and VAN Dongen, J.T. (2014). The stability and nuclear localization of the transcription factor RAP2.12 are dynamically regulated by oxygen concentration. Plant Cell Environ. http://dx.doi.org/10.1111/pce.12493.
11. Gibbs, D.J., Lee, S.C., Isa, N.M., Gramuglia, S., Fukao, T., Bassel, G.W., Correia, C.S., Corbineau, F., Theodoulou, F.L., Bailey-Serres, J., and Holdsworth, M.J. (2011). Homeostatic response to hypoxia is regulated by the N-end rule pathway in plants. Nature *479*, 415–418.
12. Licausi, F., Kosmacz, M., Weits, D.A., Giuntoli, B., Giorgi, F.M., Voesenek, L.A.C.J., Perata, P., and van Dongen, J.T. (2011). Oxygen sensing in plants is mediated by an N-end rule pathway for protein destabilization. Nature *479*, 419–422.
13. Weits, D.A., Giuntoli, B., Kosmacz, M., Parlanti, S., Hubberten, H.M., Riegler, H., Hoefgen, R., Perata, P., van Dongen, J.T., and Licausi, F. (2014). Plant cysteine oxidases control the oxygen-dependent branch of the N-end-rule pathway. Nat. Commun. *5*, 3425.
14. Gibbs, D.J., Bacardit, J., Bachmair, A., and Holdsworth, M.J. (2014). The eukaryotic N-end rule pathway: conserved mechanisms and diverse functions. Trends Cell Biol. *24*, 603–611.
15. Gibbs, D.J., Md Isa, N., Movahedi, M., Lozano-Juste, J., Mendiondo, G.M., Berckhan, S., Marín-de la Rosa, N., Vicente Conde, J., Sousa Correia, C., Pearce, S.P., et al. (2014). Nitric oxide sensing in plants is mediated by proteolytic control of group VII ERF transcription factors. Mol. Cell *53*, 369–379.
16. Hu, R.G., Sheng, J., Qi, X., Xu, Z., Takahashi, T.T., and Varshavsky, A. (2005). The N-end rule pathway as a nitric oxide sensor controlling the levels of multiple regulators. Nature *437*, 981–986.
17. Nakano, T., Suzuki, K., Fujimura, T., and Shinshi, H. (2006). Genome-wide analysis of the ERF gene family in Arabidopsis and rice. Plant Physiol. *140*, 411–432.
18. Huq, E., Al-Sady, B., Hudson, M., Kim, C., Apel, K., and Quail, P.H. (2004). Phytochrome-interacting factor 1 is a critical bHLH regulator of chlorophyll biosynthesis. Science *305*, 1937–1941.
19. Chow, K.S., Singh, D.P., Walker, A.R., and Smith, A.G. (1998). Two different genes encode ferrochelatase in Arabidopsis: mapping, expression and subcellular targeting of the precursor proteins. Plant J. *15*, 531–541.
20. Kaelin, W.G., Jr., and Ratcliffe, P.J. (2008). Oxygen sensing by metazoans: the central role of the HIF hydroxylase pathway. Mol. Cell *30*, 393–402.
21. Bailey-Serres, J., Fukao, T., Gibbs, D.J., Holdsworth, M.J., Lee, S.C., Licausi, F., Perata, P., Voesenek, L.A., and van Dongen, J.T. (2012). Making sense of low oxygen sensing. Trends Plant Sci. *17*, 129–138.
22. Miller, D.L., and Roth, M.B. (2009). C. elegans are protected from lethal hypoxia by an embryonic diapause. Curr. Biol. *19*, 1233–1237.
23. Bailey-Serres, J., and Voesenek, L.A. (2008). Flooding stress: acclimations and genetic diversity. Annu. Rev. Plant Biol. *59*, 313–339.
24. Zhong, S., Shi, H., Xue, C., Wei, N., Guo, H., and Deng, X.W. (2014). Ethylene-orchestrated circuitry coordinates a seedling's response to soil cover and etiolated growth. Proc. Natl. Acad. Sci. USA *111*, 3913–3920.
25. Kelliher, T., and Walbot, V. (2012). Hypoxia triggers meiotic fate acquisition in maize. Science *337*, 345–348.
26. Leivar, P., and Monte, E. (2014). PIFs: systems integrators in plant development. Plant Cell *26*, 56–78.
27. Ni, W., Xu, S.L., Tepperman, J.M., Stanley, D.J., Maltby, D.A., Gross, J.D., Burlingame, A.L., Wang, Z.Y., and Quail, P.H. (2014). A mutually assured destruction mechanism attenuates light signaling in Arabidopsis. Science *344*, 1160–1164.

Current Biology

Supplemental Information

Oxygen Sensing Coordinates Photomorphogenesis to Facilitate Seedling Survival

Mohamad Abbas, Sophie Berckhan, Daniel Rooney, Daniel J. Gibbs, Jorge Vicente Conde, Cristina Sousa Correia, George W. Bassel, Nora Marin-de la Rosa, José León, David Alabadí, Miguel A. Blázquez, and Michael J. Holdsworth

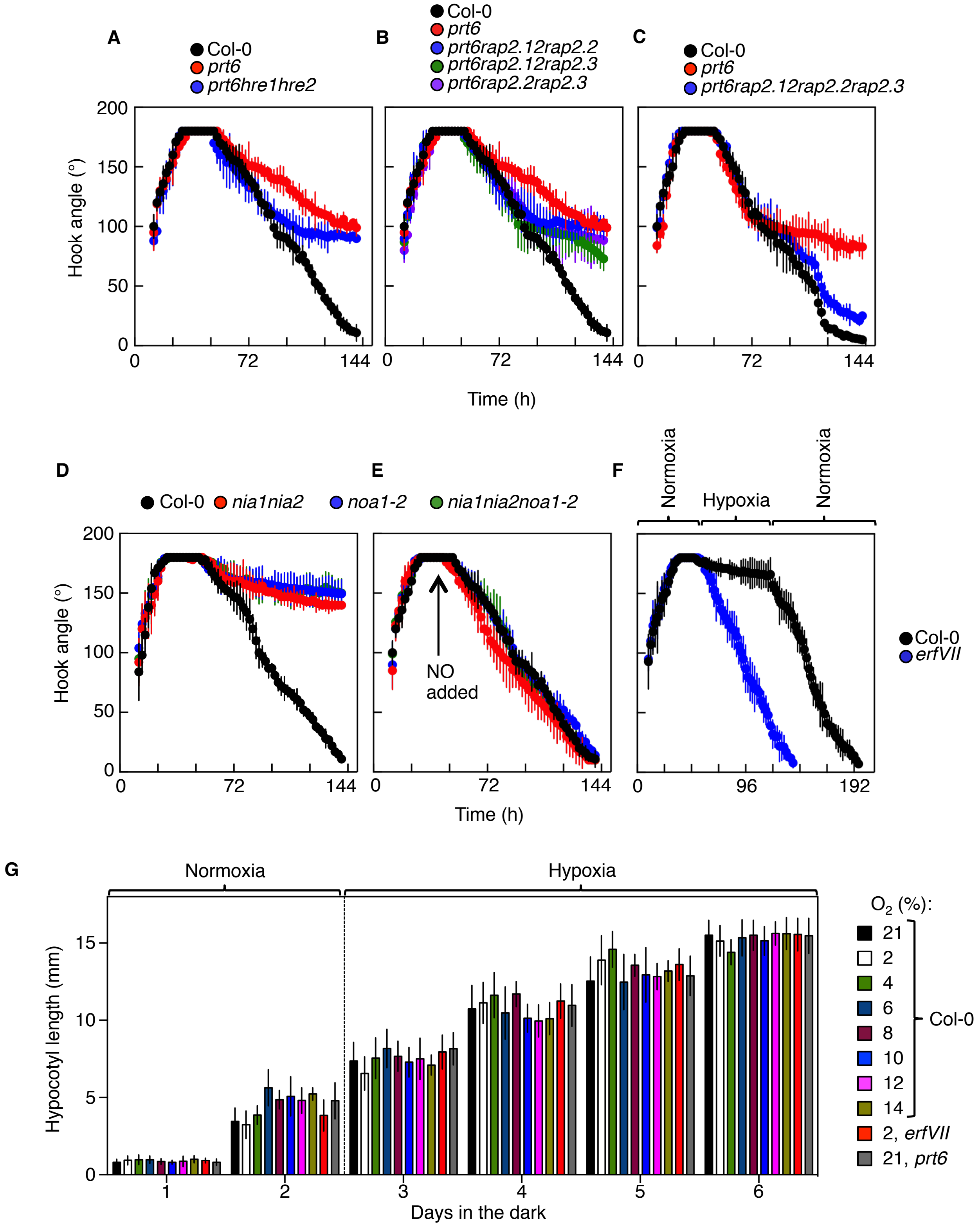
A
B
C
Col-0
prt6
prt6hre1hre2
prt6rap2.12rap2.2
prt6rap2.12rap2.3
prt6rap2.2rap2.3
prt6rap2.12rap2.2rap2.3
Hook angle (°)
200
150
100
50
0
72
144
Time (h)
D
E
F
nia1nia2
noa1-2
nia1nia2noa1-2
NO added
Normoxia
Hypoxia
Normoxia
96
192
erfVII
G
Normoxia
Hypoxia
Hypocotyl length (mm)
15
10
5
Days in the dark
1
2
3
4
5
6
O2 (%):
21
2
4
6
8
10
12
14
Col-0
2, erfVII
21, prt6

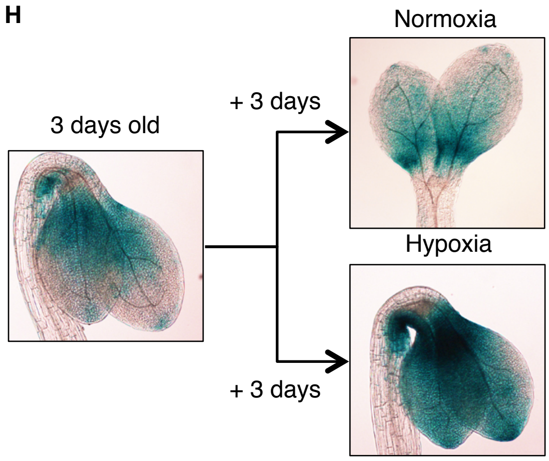

Figure S1, Related to Figure 2. Group VII ERFs function to control apical hook opening.
A,B,C. Comparison of apical hook opening of WT (Col-0), *prt6* and *prt6* in combination with increasing numbers of null mutated *erfVII* genes. The lowest order mutant combination that can open its hook is *prt6rap2.12rap2.2rap2.3*.
D. NO synthesis is required for hook opening. Mutants with reduced NO synthesis, *nia1nia2, noa1-2, nia1nia2noa1-2* all show maintenance of the apical hook in normoxia compared to WT (Col-0).
E. Application of NO gas (70ppm) removes the hook maintenance phenotype of *nia1nia2, noa1-2* and *nia1nia2noa1-2.*
F. WT (Col-0) seedling hooks remain closed during extended hypoxia but open rapidly on transfer to normoxia. *erfVII* hooks open rapidly even in hypoxia.
G. Hypocotyl elongation is not affected by hypoxia in WT and *erfVII*, or normoxia in *prt6*.
H. A gradient of auxin activity is maintained at the apical hook under hypoxia. WT transgenic seedlings expressing the auxin reporter *DR5*::*GUS* were grown for six days in normoxia or transferred after three days to hypoxic conditions. GUS staining was carried out for only four hours because longer staining resulted in very dark blue hooks.

Error bars indicate standard deviation from the mean.

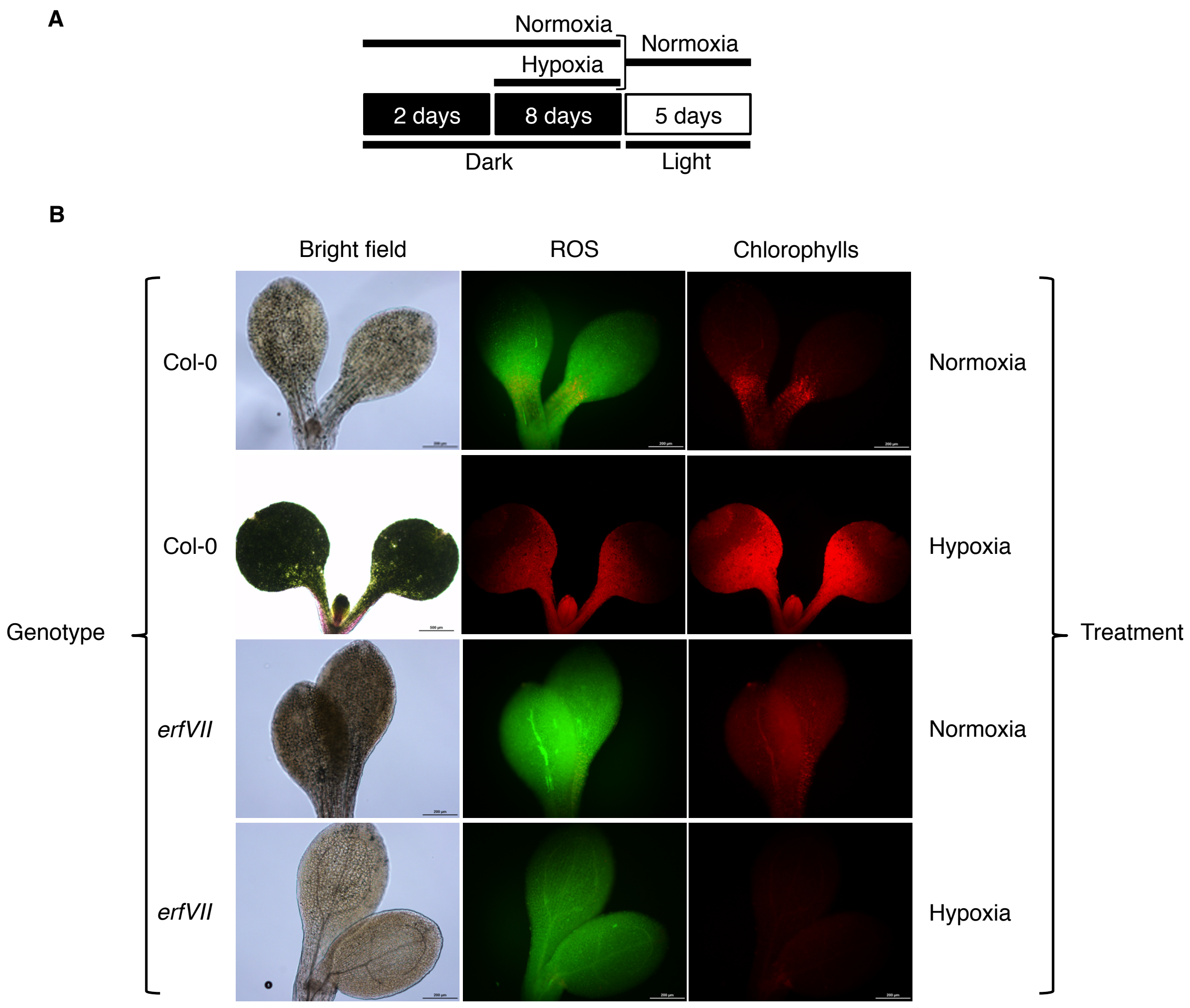

Figure S2, Related to Figure 3. Response of cotyledons to long term growth in the dark.
A. Schematic of experimental design.
B. Analysis of cotyledon phenotypes. Reactive Oxygen Species (ROS) accumulation in cotyledons is shown. Green fluorescence (H_2DCFDA staining) indicates ROS. Chlorophyll auto fluorescence is shown in red, bright field images of seedlings are shown for comparison.

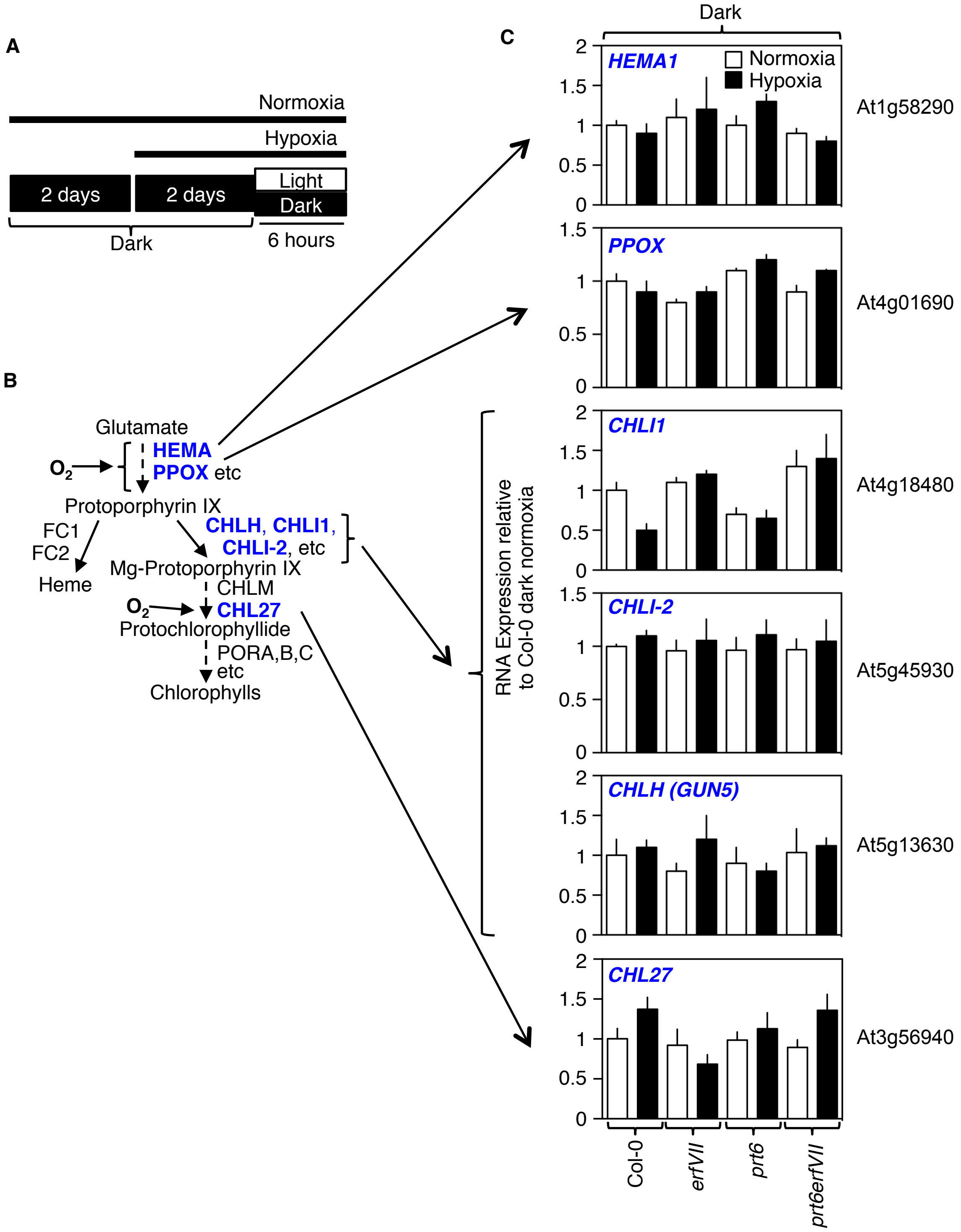
A
Normoxia
Hypoxia
2 days
2 days
Light
Dark
Dark
6 hours
B
Glutamate
O_2
HEMA
PPOX etc
Protoporphyrin IX
FC1
FC2
Heme
CHLH, CHLI1,
CHLI-2, etc
Mg-Protoporphyrin IX
CHLM
O_2
CHL27
Protochlorophyllide
PORA,B,C
etc
Chlorophylls
C
Dark
HEMA1
Normoxia
Hypoxia
At1g58290
PPOX
At4g01690
CHLI1
At4g18480
CHLI-2
At5g45930
CHLH (GUN5)
At5g13630
CHL27
At3g56940
RNA Expression relative
to Col-0 dark normoxia
Col-0
erfVII
prt6
prt6erfVII

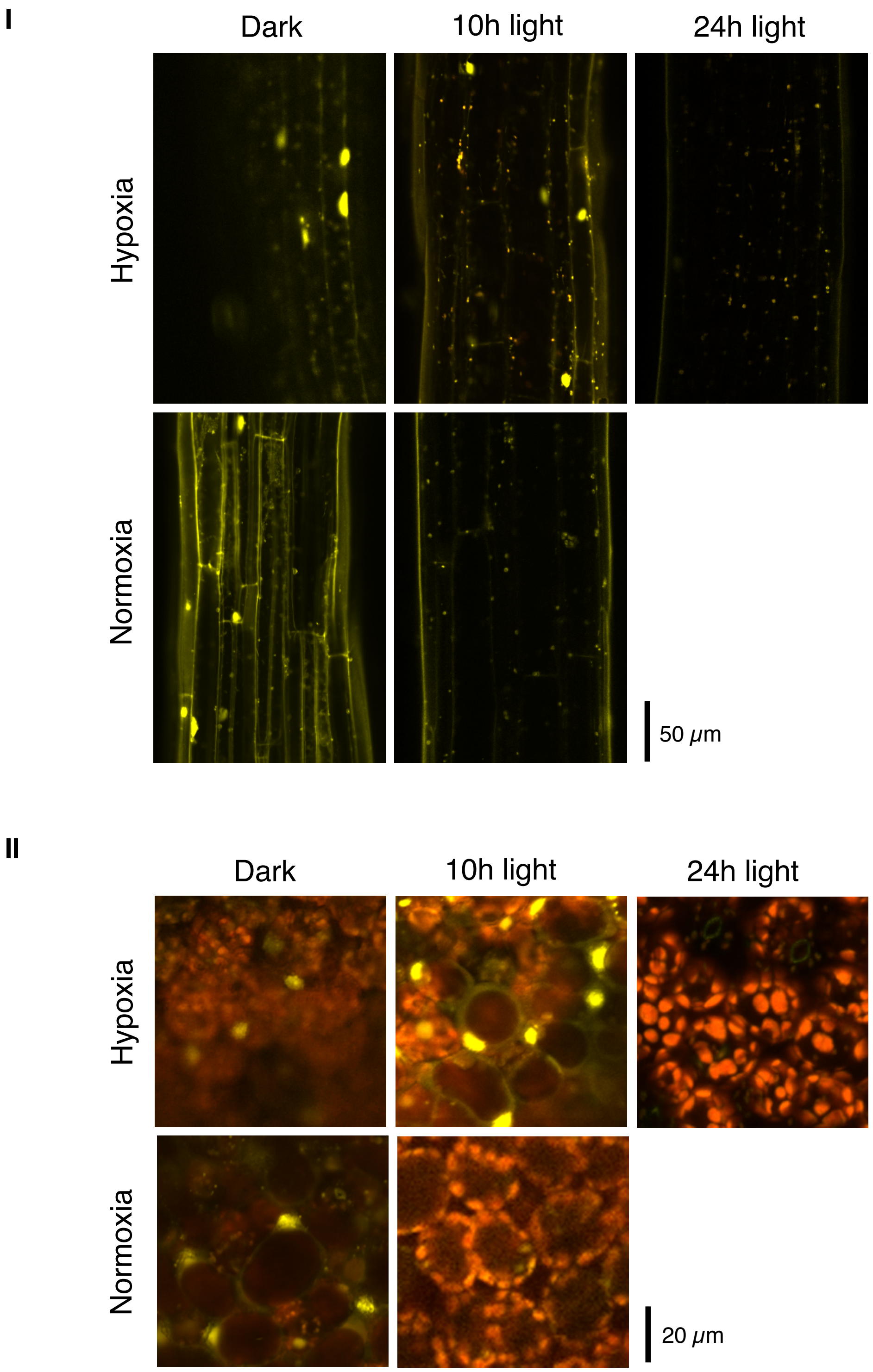

Figure S3, Related to Figure 4. Expression of many tetrapyrrole biosynthesis genes is not affected by hypoxia, and light and hypoxia influence ERFVII subcellular location Quantitative (Q)rtPCR analysis of chlorophyll biosynthesis gene expression in etiolated seedlings of WT (Col-0) and N-end rule pathway mutants grown in normoxia or hypoxia.

A. Experimental setup. Etiolated seedlings were grown for four days in the dark, the final 2 days being in either normoxia or hypoxia, then seedlings were left in the dark (this figure) or exposed to light (Figure 4) for 6 hours.

B. Schematic of the chlorophyll biosynthesis pathway in higher plants. C. QrtPCR of selected genes from 4 day old etiolated seedlings, the final 2 days being in either normoxia or hypoxia.

D. Hypoxia causes delayed in light-regulated removal of nuclear located YFP-RAP2.3 (*35S:YFP-RAP2.3*)

in (I) hypocotyls and (II) cotyledons.

Error bars indicate standard deviation from the mean.

Supplemental Experimental Procedures:

Plant material:

Unless otherwise stated *Arabidopsis thaliana* seeds were obtained from the Nottingham *Arabidopsis* Stock Centre (NASC) and were grown for propagation as previously described [S1]. All mutants are in the Col-0 (Wild Type) accession. Novel reported mutant combinations were identified by PCR (genotyping primers as previously described [S1]). White poppy (*Papaver somniferum* L.) and *Nicotiana benthamiana* Domin. seeds were obtained from Cristina Ferrándiz (IBMCP, Valencia, Spain). A *pENTR* vector containing the *RAP2.3* ORF has been described [S2]. To prepare *35S:YFP-RAP2.3*, the *RAP2.3* entry clone was transferred by a LR reaction to the destination vector *pEarleyGate104* [S3] using the Gateway technology (Invitrogen). Transgenic *Arabidopsis* containing *promERFVII:MA-ERFVII* constructs, using full-length genomic DNA sequence (2 kb upstream of the ATG, finishing at the STOP codon) as PCR template, were produced as previously described for *promRAP2.3:MA-RAP2.3* [S1] using primers described in Table S1, and transformed into *Agrobacterium* (strain GV3101 pMP90) and *Arabidopsis* using standard protocols *[S4]*.

Analysis of seedling growth:

Seeds were plated on half-strength Murashige and Skoog (MS) medium (Duchefa) with 1% sucrose, 1% agar (pH 5.7), chilled for 2 to 4 days at 4°C, and exposed to white fluorescent light (90–100 μmol m^{-2} sec^{-1}) for 8-12 hours at 22°C to initiate germination, after which plates were incubated in darkness, unless indicated in the text. Experiments analyzing the effect of different oxygen levels were conducted as follows; seedlings were placed 48 hours after germination in a transparent

methacrylate container (volume = 22 l) connected to a nitrogen (N_2) cylinder (Linde Industrial Gases, Spain) and an oxygen meter attached to the outlet pipe (KANE250 Compact Flue Gas Analyzer-Kane International Ltd, UK). Nitrogen was flushed into the system until the desired oxygen level was reached, and oxygen levels were monitored at the beginning and end of the experiment. For NO treatments, 70 ppm (final concentration) pure NO (Linde Industrial Gases, Spain) was injected into the container. For analysis of Reactive Oxygen Species (ROS) seedlings were grown in the dark in normoxia or hypoxia and moved into continuous white light (95 µmol m^{-2} s^{-1}) for an additional 4 d. Seedlings were incubated for 30 min at 4°C in 10 µM H_2DCFDA (a ROS-sensitive dye) and then washed with 10 mM MES, 0.1 mM CaCl2, pH 6, for 60 min at 22°C. Dye excitation was at 480 nm; emitted light was detected at 535 to 550 nm with a Nikon Eclipse E600 [S5, 6].

Time-course analysis of apical hook development

Development of seedlings was recorded at 1-hour intervals for 7 days from transfer to dark at 22°C with an infrared light source (850 nm LED) by a spectrum-enhanced camera (Microsoft lifestudio). Angles between the hypocotyl axis and cotyledons were measured with ImageJ (NIH; http://rsb.info.nih.gov/ij) as previously reported [S7]. Fifteen seedlings with synchronized germination were processed. Experiments were repeated at least twice.

Gene expression analysis

RNA extraction, cDNA synthesis, and quantitative RT-PCR (RT-qPCR) have been previously described [S8]. Gene-specific signals were normalized over those of the *EF1a* (AT5G60390). For primers used see table S1. In the case of DR5::GUS, β-

glucuronidase (GUS) staining was performed as described previously [S9]. Seedlings were collected and fixed with 90% cold acetone, washed twice with a solution of 50 mM NaPO4 (pH 7.2), 0.2 % (v/v) Triton X-100 (Sigma), 2 mM Potassium Ferrocyanide and Potassium Ferricyanide (Sigma), then 1 mM X-Gluc (Sigma) was added and seedlings were stained overnight at 37°C then cleared with a series of ethanol washes and with chloral hydrate (Sigma) for 2 days. Images were taken using a Nikon Eclipse E600 scope.

Confocal microscopy

Fresh seedlings were mounted on slides with water. Images were taken using a Zeiss LSM 780 confocal microscope on an Axio Observer (Zeiss, http://www.zeiss.com) with a detection wavelength of 497-532 nm.

Analysis of chlorophyll and protochlorophyllide

Four-day-old dark-grown seedlings were exposed to white light for 6 h of and chlorophyll was extracted in darkness from 100 mg tissue with 1 ml of acetone (80%) at 4°C. The chlorophyll content was calculated using spectrophotometric absorbance (*A*) at wavelengths of 603, 645 and 663 nm and 80% acetone as a control, and shown as milligram of chlorophyll per gram of fresh tissue as follows: Chlorophyll a (mg g^{-1}) = $12.7 \times A_{663} - 2.69 \times A_{645}$; chlorophyll b (mg g^{-1}) = $22.9 \times A_{645} - 4.86 \times A_{663}$: and chlorophyll a+b (mg g^{-1}) = $8.02 \times A_{663} + 20.20 \times A_{645}$. Protochlorophyllide levels were measured as previously described [S10, 11]. Twenty dark-grown seedlings were homogenized in 0.8 ml of ice-cold 90% acetone and 0.1% ammonia, left on ice in darkness for 6 hours, centrifuged for 20 minutes at 4°C, and then relative fluorescence

(excitation at 440 nm; emission between 600-700 nm) was measured at room temperature.

Supplemental References:

S1. Gibbs, D.J., Isa, N.M., Movahedi, M., Lozano-Juste, J., Mendiondo, G.M., Berckhan, S., Marin-de la Rosa, N., Conde, J.V., Correia, C.S., Pearce, S.P., et al. (2014). Nitric Oxide Sensing in Plants Is Mediated by Proteolytic Control of Group VII ERF Transcription Factors. Molecular Cell *53*, 369-379.

S2. Marin-de la Rosa, N., Sotillo, B., Miskolczi, P., Gibbs, D.J., Vicente, J., Carbonero, P., Onate-Sanchez, L., Holdsworth, M.J., Bhalerao, R., Alabadi, D., et al. (2014). Large-Scale Identification of Gibberellin-Related Transcription Factors Defines Group VII ETHYLENE RESPONSE FACTORS as Functional DELLA Partners. Plant Physiol. *166*, 1022-1032.

S3. Earley, K.W., Haag, J.R., Pontes, O., Opper, K., Juehne, T., Song, K.M., and Pikaard, C.S. (2006). Gateway-compatible vectors for plant functional genomics and proteomics. Plant J. *45*, 616-629.

S4. Clough, S.J., and Bent, A.F. (1998). Floral dip: a simplified method for *Agrobacterium*-mediated transformation of *Arabidopsis* thaliana. Plant J. *16*, 735-743.

S5. Achard, P., Renou, J.-P., Berthome, R., Harberd, N.P., and Genschik, P. (2008). Plant DELLAs restrain growth and promote survival of adversity by reducing the levels of reactive oxygen species. Current Biology *18*, 656-660.

S6. Cheminant, S., Wild, M., Bouvier, F., Pelletier, S., Renou, J.-P., Erhardt, M., Hayes, S., Terry, M.J., Genschik, P., and Achard, P. (2011). DELLAs Regulate Chlorophyll and Carotenoid Biosynthesis to Prevent Photooxidative Damage during Seedling Deetiolation in Arabidopsis. Plant Cell *23*, 1849-1860.

S7. Gallego-Bartolome, J., Arana, M.V., Vandenbussche, F., Zadnikova, P., Minguet, E.G., Guardiola, V., Van der Straeten, D., Benkova, E., Alabadi, D., and Blazquez, M.A. (2011). Hierarchy of hormone action controlling apical hook development in Arabidopsis. Plant J. *67*, 622-634.

S8. Frigerio, M., Alabadi, D., Perez-Gomez, J., Garcia-Carcel, L., Phillips, A.L., Hedden, P., and Blazquez, M.A. (2006). Transcriptional regulation of gibberellin metabolism genes by auxin signaling in arabidopsis. Plant Physiol. *142*, 553-563.

S9. Zadnikova, P., Petrasek, J., Marhavy, P., Raz, V., Vandenbussche, F., Ding, Z., Schwarzerova, K., Morita, M.T., Tasaka, M., Hejatko, J., et al. (2010). Role of PIN-mediated auxin efflux in apical hook development of Arabidopsis thaliana. Development *137*, 607-617.

S10. Goslings, D., Meskauskiene, R., Kim, C.H., Lee, K.P., Nater, M., and Apel, K. (2004). Concurrent interactions of heme and FLU with Glu tRNA reductase (HEMA1), the target of metabolic feedback inhibition of tetrapyrrole biosynthesis, in dark- and light-grown Arabidopsis plants. Plant J. *40*, 957-967.

S11. Zhong, S.W., Shi, H., Xue, C., Wei, N., Guo, H.W., and Deng, X.W. (2014). Ethylene-orchestrated circuitry coordinates a seedling's response to soil cover and etiolated growth. PNAS (USA) *111*, 3913-3920.
